# Repeated clear benefits of immunotherapy in a patient with Charcot-Marie-Tooth disease carrying a rare point mutation in *PMP22*

**DOI:** 10.1007/s10048-025-00808-9

**Published:** 2025-03-24

**Authors:** Honami Kawai, Yoichiro Nishida, Takashi Kanda, Takanori Yokota

**Affiliations:** 1 Department of Neurology and Neurological Science, Graduate School of Medical and Dental Sciences, Institute of Science Tokyo, 1-5-45, Yushima, Bunkyo-Ku, Tokyo, 113-8519 Japan; 2 Department of Neurology and Clinical Neuroscience, Yamaguchi University Graduate School of Medicine, Yamaguchi, 755-8505 Japan

**Keywords:** Charcot-Marie-Tooth disease (CMT), PMP22, Point mutation, Chronic inflammatory demyelinating polyneuropathy (CIDP), Optic neuritis

## Abstract

We describe a unique patient who had been diagnosed with inflammatory demyelinating polyneuropathy (CIDP) for 13 years with frequent clear responses to immunotherapies and was finally diagnosed with Charcot-Marie-Tooth disease (CMT) with a rare point mutation in PMP22 (c.320G > A, p.G107D). Some patients diagnosed with young-onset CIDP may have underlying CMT, and extensive genetic testing including point mutations of PMP22 gene is required not to miss the diagnosis.

## Introduction

The peripheral myelin protein-22 (PMP22) duplication is the most common cause of Charcot-Marie-Tooth disease (CMT) [1, 2]. In addition, several point mutations in PMP22 have been identified to be associated with the disease [2]. Only one report has described CMT patients with a missense mutation in PMP22 (c.320G > A, p.G107D), who showed typical demyelinating CMT features [2]. Here, we report a unique case of CMT with this rare missense mutation, initially presenting with features suggestive of chronic inflammatory demyelinating polyneuropathy (CIDP).

## Case presentation

**Fig. 1:**
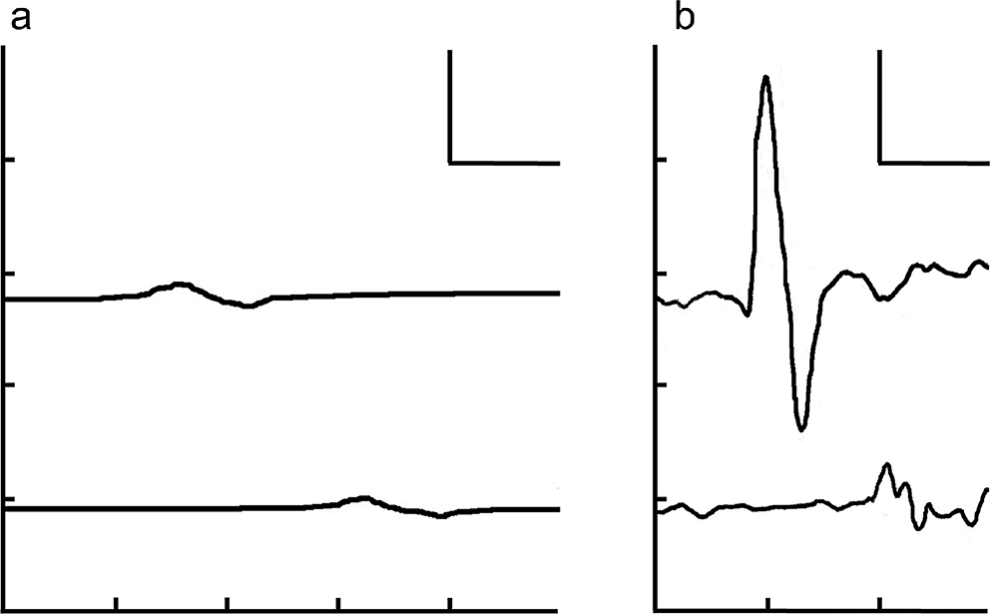
NCS of the right median nerve before and after immunotherapies at 9 years of age. In each panel, the upper and lower traces show the CMAPs recorded on the abductor pollicis muscle, which were evoked by supramaximal stimulations at the wrist and the elbow, respectively. The CV in the forearm segment was severely impaired (12.4 m/s), TL was markedly prolonged (11.6 ms), and the distal CMAP amplitude was decreased (0.79 mV) before the treatment (**a**). After the immunotherapies, CV, TL, and distal CMAP amplitude improved to 16.2 m/s, 8.28 ms, and 6.27 mV, respectively (**b**). Scale bars represent 10 ms for time and 2 mV for amplitude. CMAP, Compound muscle action potential; CV, Conduction velocity; NCS, Nerve conduction study; TL, Terminal latency

A 22-year-old woman with no family history of neuromuscular disease was referred to our hospital for the evaluation and treatment of peripheral neuropathy. Her developmental milestones were not delayed until approximately one year old. At two years of age, parents noticed frequent falls. At 8 years of age, she experienced acute deterioration of vision in both eyes, with a bilateral reduced visual acuity of 0.6. Grip strength weakened, and her handwriting became clumsy. Her gait worsened, and she could not stand or climb stairs without assistance. She also developed distal dominant symmetric paresthesia in all four limbs. The patient visited the hospital at 9 years of age. Neurological examination revealed distal dominant limb weakness, distal calf muscle atrophy, glove and stocking sensory impairment, four limbs areflexia, and bilateral pes cavus. The nerve conduction study (NCS) of median nerves showed decreased compound muscle action potential (CMAP) amplitudes, severe slowing of conduction velocities (CV) in the forearm segment, and markedly prolonged terminal latencies on both sides (Table 2; Fig. 1a). Sensory nerve action potentials (SNAPs) of the median and sural nerves and CMAPs of the tibial nerve were not detected. Both cell count (8 /µL, monocyte 100%) and protein level (1.27 g/L) were elevated in the cerebrospinal fluid (CSF). Fluorescence in situ hybridization (FISH) analysis for detecting the PMP22 gene duplication was negative. Due to the negative DNA test result and markedly elevated CSF protein level (> 1 g/L) [3], despite the presence of foot deformities and early-onset demyelinating neuropathy, chronic inflammatory demyelinating polyneuropathy (CIDP) rather than CMT was suspected. Intravenous immunoglobulin (IVIg) and high-dose intravenous methylprednisolone (IVMP) pulse therapy, followed by oral prednisone (PSL) at 35 mg/day (1 mg/kg/day) were initiated. She was able to climb stairs without assistance, and the paresthesia disappeared. In the follow-up NCS, the distal CMAP amplitude of the right median nerve increased to 6.27 mV, indicating the resolution of the conduction block at the most distal segment (Fig. 1b). Because of this marked improvement of NCS findings, we were able to completely deny the possibility that her clinical improvement was only due to the placebo effect of immunotherapies. The clear effect of immunotherapies on neurological and electrophysiological examinations finally led to the diagnosis of CIDP. Her visual acuity also improved to 1.2 bilaterally. Visual evoked potentials (VEPs) after the recovery of her vision still showed prolonged P100 latencies bilaterally (114 ms on the left and 110 ms on the right [normal upper limit; 109 ms]). Although no evidence of inflammation in the optic nerve was shown in her brain MRI, apparent recovery of visual acuity after immunotherapies may have suggested optic neuritis associated with CIDP. Monthly IVIg therapy was continued, and the daily dose of PSL was gradually decreased to 10 mg. Two weeks after the PSL dosage reached 10 mg/day, she developed limb weakness and paresthesia again and became bound to a wheelchair. IVMP followed by an increase of the daily PSL dose to 30 mg enabled her to walk independently again. Subsequently, 120 mg/day of cyclosporine was administered, and the PSL dosage was gradually reduced. Maintenance therapy with IVIg was continued every two months. Her distal motor weakness did not fully improve, and she began to use ankle–foot orthoses at 10 years of age. Over the following several years, until the age of 19 years, whenever the dosage of PSL reached less than 10 mg/day, she experienced several exacerbations of limb weakness predominantly on the right side, which were partially relieved by IVIg or increased PSL dosage. Cervical and lumbosacral MRI revealed nerve root enlargement without abnormal gadolinium enhancement. At the age of 19 years, the monthly IVIg was switched to subcutaneous immunoglobulin (SCIg) therapy (0.24 g/kg/week). PSL was gradually tapered because her symptoms were relatively stable. Within a month after the PSL dose was reduced to 12 mg/day, she noticed deterioration of her gait and was referred to our hospital for reevaluation.

**Table 1:** Electrophysiological findings

Nerve	Stimulation site	Latency (ms)	Amplitude (mV)	Velocity (m/s)
Right median nerve	Wrist	11.6	0.79	
				12.4
	Elbow	25.4	0.60	
Left median nerve	Wrist	11.6	0.79	
				13.8
	Elbow	23.6	0.59	

Neurological examination revealed generalized areflexia, bilateral pes equinus, pes cavus, and hammer toes with distal lower extremity atrophy. Her lower extremities showed asymmetrically reduced manual muscle test (MMT) grades, which were prominent in the distal part and right side (Table 2). The muscle strength of the upper extremities was relatively preserved, although her hand muscles were grade 4 on the MMT. Touch and pain sensations were impaired in a glove-and-stocking manner. Neither cell count nor protein level was raised, and oligoclonal bands were absent in the CSF. Anti-ganglioside and anti-neurofascin 155 antibodies were negative in the serum. Motor NCS showed homogenous slowing without temporal dispersion or conduction block, suggesting secondary axonal loss due to demyelinating polyneuropathy. Ultrasound examination revealed enlargement of the cervical nerve roots and diffuse homogeneous hypertrophy in the entire length of the ulnar and median nerves. There were no abnormalities in the VEPs, fundus examination and optical coherence tomography, and the brain MRI showed no signs of optic neuritis. Although the medical history of the effectiveness of immunotherapy suggested that her neuropathy was an inflammatory one, all results obtained in our hospital were suggestive of CMT. Genetic testing revealed a heterozygous missense mutation in PMP22 (c.320G > A, p.G107D) that was reported to cause a classic demyelinating CMT phenotype [2].

**Table 2:** Manual muscle test (MMT) grades of lower limbs

Muscle	MMT grade (right/left)
Iliopsoas	3-/3
Gluteus	3/4
Quadriceps	2/3
Hamstrings	2/3
Tibialis anterior	0/2
Gastrocnemius	1/2

## Discussion

Various studies have suggested an association between CMT and inflammatory neuropathy in response to immunotherapy [3]. The diagnosis of CMT may be challenging in the presence of inflammatory features or when a family history is lacking [4]. While nerve root thickening is a shared radiological finding between CMT and CIDP [3], the diameter of the peripheral nerves is homogeneously larger in CMT than in CIDP [4]. Therefore, diffuse homogeneous hypertrophy of the entire nerve, as seen in our patient, is a red flag that prompts genetic workup for CMT [4].

Our patient with genetically confirmed CMT showed CIDP-like presentations, such as a relapsing and remitting course, positive sensory symptoms, proximal weakness, and a markedly elevated CSF protein concentration above 1 g/L at the first visit. These clinical presentations are consistent with the overlapping features of CMT and inflammatory peripheral neuropathy [3]. In addition, the optic nerve was also impaired in our patient, possibly suggesting that inflammatory demyelination occurred in the central nervous system as seen in a few CMT cases with PMP22 duplication [5, 6]. However, no CMT patients with point mutations in the PMP22 gene have been reported to bear inflammatory features to date.

The pathophysiology of CMT with inflammatory features remains unknown [3]. However, there was a trend towards a higher proportion of anti-PMP22 antibodies in a subset of 12 patients with stepwise progression from a cohort of 55 participants with CMT1A due to PMP22 duplication [3]. Moreover, peripheral T-cell responses to various myelin epitopes in a case of the coexistence of optic neuritis and CMT with duplication of the PMP22 gene were reported to show highly significant proliferation against the CNS myelin protein, proteolipid protein (PLP), which shares partial homology with PMP22 [5]. Taken together, while the coexistence of the inflammatory and hereditary neuropathies may be coincidental, we speculate that some immunological mechanism could link them in our case.

## Conclusion

We report the first patient with CMT caused by a rare missense PMP22 mutation presenting with clinical features suggestive of CIDP. Some patients diagnosed with young-onset CIDP may have underlying CMT. Not only duplication but also point mutations of the PMP22 gene should be analyzed when coexistence of CMT and inflammatory neuropathy is suspected.

## Data Availability

No datasets were generated or analysed during the current study.
